# The use of randomisation-based efficacy estimators in non-inferiority trials

**DOI:** 10.1186/1745-6215-16-S2-P129

**Published:** 2015-11-16

**Authors:** David Gillespie, Kerenza Hood, Daniel Farewell, Antony Hawthorne, Chris Probert, Rachel Stenson, Peter Barrett-Lee, Angela Casbard, Nick Murray

**Affiliations:** 1 Cardiff University, Cardiff, UK; 2 Cardiff and Vale University Health Board, Cardiff, UK; 3 University of Liverpool, Liverpool, UK; 4 Royal Adelaide Hospital, Adelaide, Australia

## 

The goal of a non-inferiority (NI) trial is to determine whether or not a new treatment is no worse than an existing treatment on some outcome of interest. Analysis based on the intention-to-treat (ITT) principle is anti-conservative in this instance, so current guidelines recommend analysing on a per-protocol (PP) population in addition. However, PP analysis relies on the often implausible assumption of no unmeasured confounders. Randomisation-based efficacy estimators (RBEEs) allow for treatment non-adherence whilst maintaining a comparison of randomised groups. Fischer et al. have developed an approach for estimating RBEEs in randomised trials with two active treatments, a common feature of NI trials.

The aim of this talk is to demonstrate the use of these methods, proposing their use as a primary analysis in NI trials.

By treating randomisation as an instrument, estimates of treatment efficacy can be derived that maintain a comparison of randomised groups and are therefore not prone to selection bias. These models can be fitted using instrumental variables regression using standard statistical software. In addition, the method relies on identifying baseline variables that differentially predict adherence by treatment group, while remaining independent of outcome.

Two examples of real NI trials are presented; where it is and is not possible to derive distinct causal estimators. The process of identifying predictors will be described, and the estimates of treatment efficacy will be presented alongside the ITT and PP analyses. The process of estimating treatment efficacy when distinct causal parameters cannot be identified will also be demonstrated.

This work will highlight the value of this approach when estimating treatment effects in NI trials. It will also highlight the importance of collecting baseline data that may be associated with adherence differently in the different treatment groups, and also the how adherence to study treatment is collected and defined.

